# Modeling of the Longitudinal Torsional Ultrasonic Vibration-Assisted Milling of UD-CF/PPS Composites Through Capturing the Influences of Both the Longitudinal and the Torsional Vibrations

**DOI:** 10.3390/mi17080881

**Published:** 2026-07-24

**Authors:** Jiawei Mei, Jin Tian, Huanzong Ke, Yikang Liang

**Affiliations:** 1 School of Mechanical, Electronic and Control Engineering, Beijing Jiaotong University, Beijing 100044, China; jwmei@bjtu.edu.cn (J.M.);; 2 Key Laboratory of Vehicle Advanced Manufacturing, Measuring and Control Technology, Ministry of Education, Beijing Jiaotong University, Beijing 100044, China

**Keywords:** CF/PPS, ultrasonic vibration-assisted machining, longitudinal torsional vibration

## Abstract

Owing to its infinite shelf-life under ambient conditions, satisfactory recyclability, and great reparability, carbon fiber-reinforced polyphenylene sulfide (CF/PPS) has been increasingly applied in the near-net-shape manufacture of high-value components. Longitudinal torsional ultrasonic vibration-assisted milling (LTUVAM) shows strong potential as an advanced processing technology for the efficient precision machining of composites. However, studies and models on the explanation of LTUVAM of CF/PPS composites seem to be missing in the literature. This paper proposes a finite element analysis method for LTUVAM of UD-CF/PPS processes. The kinematic analysis of the LTUVAM is proposed first, then the mechanism of surface formation during UD-CF/PPS milling process is provided. A simulation method which could simultaneously achieve both longitudinal and torsional vibration motions is introduced in the finite element model, enabling the simulation of LTUVAM of UD-CF/PPS composites. The experimental validations were conducted under both CM and LTUVAM conditions with three different cutters, demonstrating that cutting force simulations have significant agreement with experimental data, and both simulation and experiment indicate that LTUVAM produces superior surface quality compared to CM; the fiber debonding at the microscopic level could be eliminated and the height of machined surfaces could be significantly reduced when LTUVAM is utilized. These findings could also open avenues for clarification of other scientific queries such as cutting parameters optimization, cutting tool selection, and modeling of the UD-CF/PPS drilling process.

## 1. Introduction

Carbon fiber-reinforced polymers (CFRPs) can be generally classified into two categories based on the matrix type: carbon fiber-reinforced thermosetting composites (CFRTSs) and carbon fiber-reinforced thermoplastic composites (CFRTPs) [1]. Owing to their superior mechanical properties and significant lightweight advantages [2,3,4,5], CFRTSs have become critical materials in fields such as aerospace, high-end equipment, and automotive manufacturing. Compared to CFRTSs, CFRTPs could not only offer higher specific strength and better heat resistance but also boast remarkable benefits in terms of storage conditions, molding cycles, and energy consumption. Specifically, CFRTPs require less stringent storage environments, significantly shorten forming cycles, and substantially reduce energy consumption during production [6,7]. Most notably, the thermoplastic matrix endows CFRTPs with excellent recyclability and convenient repairability [8,9]. Based on their mechanical and thermal properties, CFRTPs can be systematically classified into three major categories: high-performance polymers, engineering polymers, and standard polymers [10]. Due to their outstanding advantages in stiffness and strength, CFRTPs have emerged as core supporting materials across various fields, including automotive, aerospace, and new energy.

As a new generation of high-performance thermoplastic composites, carbon fiber-reinforced polyphenylene sulfide (CF/PPS) has been widely applied in the manufacturing of structural components for aerospace vehicles and transportation equipment. This is attributed to its excellent mechanical properties, chemical resistance, radiation resistance, and reprocess ability [11,12,13,14]. However, since carbon fibers are hard and brittle while PPS is a ductile thermoplastic material, the combination of such high-strength fibers and a tough matrix makes CF/PPS a typical difficult-to-machine material, presenting numerous challenges during machining processes. Due to its pronounced anisotropy and low interlaminar strength, the composite is prone to defects such as delamination and burrs under cutting forces [15]. Meanwhile, the cutting heat generated during machining cannot be effectively dissipated, leading to the thermal degradation of the resin matrix and severely compromising the service performance and lifespan of the components.

Traditional machining methods often struggle to balance processing efficiency and quality requirements when machining CF/PPS composites, as they inevitably generate high cutting forces, elevated cutting temperatures, and severe cutting vibrations during the process. In this context, the development of ultrasonic-assisted technology offers an alternative approach for machining CF/PPS composites. As a novel hybrid machining process, ultrasonic-assisted machining (UAM) [16] enhances conventional machining by applying high-frequency ultrasonic vibrations. By superimposing micro-vibrations onto traditional cutting motions, UAM alters the contact state between the tool and the workpiece, demonstrating tremendous potential in the field of difficult-to-machine materials.

Studies have demonstrated that ultrasonic vibration-assisted machining could significantly improve cutting performance, reduce cutting forces and tool wear, and enhance machined surface quality. Introducing ultrasonic-assisted technology into the milling process of CF/PPS composites holds great promise for resolving the issues associated with conventional machining methods. However, due to the unique microstructure and mechanical behavior of CF/PPS composites, the underlying mechanisms of their ultrasonic-assisted machining have not yet been fully elucidated. Consequently, there is a lack of modeling of the ultrasonic-assisted machining of CF/PPS composites.

Most studies on the machining of CFRTP materials have focused on the carbon fiber-reinforced polyetheretherketone (CF/PEKK) materials. Guo et al. [17] investigated the effects of spindle speed and feed rate on cutting forces, cutting temperatures, chip morphology, and hole quality during the helical milling of CF/PEEK composites. Their study revealed that the axial force is primarily governed by the feed rate and exhibits a dynamic attenuation trend due to the thermal softening effect. Liu et al. [18] investigated the milling performance and residual mechanical behavior of CF/PEEK and conventional CFRP composites. They found that due to the ductile matrix and strong interfacial bonding with fibers, CF/PEEK composites exhibit superior machining quality compared to conventional CFRPs. Meng et al. [19] compared the drilling characteristics of unidirectional conventional CFRP and CF/PEEK composites under various parameters. Their research illustrated that, owing to the higher ductility and toughness of PEEK, CF/PEEK generates continuous chips during drilling, accompanied by higher drilling temperatures, greater feed thrust, and smaller hole damage areas.

Researchers also utilized the finite element analysis (FEA) model for CF/PEEK machining research. Takayu et al. [20] proposed a FEA method which could simulate fiber-dominated and matrix-dominated damage modes separately by accounting for the interaction between these two damage modes; this approach enabled a more refined and comprehensive progressive damage analysis. Liu et al. [21] developed a three-dimensional braided finite element model considering anisotropic heat transfer characteristics to predict the machining quality of CFRTPs. Ge et al. [22] proposed a new micro-scale FEA model that incorporated the thermal softening effects of both the matrix and the interface; this model clarified the relations among the stiffness of the thermoplastic matrix, interfacial bonding strength, fracture toughness, and temperature during the orthogonal cutting of CF/PEEK. Bao et al. [23] established a three-dimensional micro-scale FEA model for unidirectional CF/PEEK milling to analyze the effects of various parameters on fiber-matrix interfacial damage by applying diverse loading conditions to structural representative volume elements; they investigated how interfacial damage and variations in crystallinity affect the macroscopic elastic properties of UD-CF/PEEK. Their findings indicated that the mechanical properties of the machined surface are significantly influenced by machining-induced interfacial damage and changes in crystallinity.

To improve the machining behavior and surface finish, the ultrasonic vibration-assisted technology was introduced in the machining of CF/PEEK materials. Zhang et al. [24] proposed a machining method involving simultaneous vibrations in both the feed and the vertical directions and provided the optimal milling parameters to reduce cutting forces. Additionally, Zhang et al. [25] designed and manufactured a large-amplitude longitudinal ultrasonic vibration-assisted milling (LUVAM) tool holder to increase the clearance between the tool and the workpiece; hence, cutting forces were reduced and the chip removal efficiency was increased. Liu et al. [26] investigated the material removal mechanisms during UVAM of CF/PEEK and discussed the effect of the fiber cutting angle on the machining performance. Hiromitsu et al. [27] applied ultrasonic vibrations to needle-shaped tools contacting CFRTPs to soften the thermoplastic matrix resin. Zhang et al. [28] established a three-dimensional thermo-mechanical coupled finite element model considering ultrasonic frequency, amplitude, and fiber orientation angles to simulate the CFRP cutting process, illustrating that the ultrasonic vibration-assisted machining operation significantly improves surface integrity and reduces cutting forces.

Most of the studies in the UAM of CFRTPs only focused on the unidirectional longitudinal ultrasonic vibration method; the longitudinal torsional ultrasonic vibration technology, as a more advanced machining method, has only been studied by a few researchers. Wang et al. [29] utilized the longitudinal torsional ultrasonic vibration-assisted milling (LTUVAM) method to cut unidirectional CF/PEEK plates, indicating that compared to CM operation, LTUVAM reduced cutting forces by 4–54.1% and surface roughness by 15.8–66.9%. However, this study has not given any model or method to understand and simulate the LTUVAM process. Furthermore, all the ultrasonic vibration-assisted machining research works mentioned above focused on the CF/PEEK composites. The research of longitudinal torsional ultrasonic vibration technology in the milling of CF/PPS materials is lacking; an approach which could be utilized to simulate the effects of longitudinal torsional ultrasonic vibration on cutting forces and machined surface integrity is required.

To address this research gap, a FEA model for the LTUVAM of CF/PPS composites is developed in this paper. To implement the longitudinal torsional ultrasonic vibration motion in finite element simulations, a kinematic analysis of the LTUVAM is proposed first, then a strategy which could calculate the amplitudes of the tool’s axial displacement and torsional angle at any instance is provided to reflect the tool’s longitudinal and torsional vibrations. The mechanism of surface formation and a finite element model for CF/PPS milling process are then presented, enabling the simulation for the LTUVAM of CF/PPS. Three types of milling cutter are utilized to validate the presented method, and the effectiveness of the LTUVAM on cutting force reduction and machined surface quality improvement has also been investigated.

In the following sections of this article, Section 2 presents the mechanism of the LTUVAM of UD-CF/PPS composites, which becomes the foundation for finite element modeling of the LTUVAM of UD-CF/PPS (provided in Section 3). Then, Section 4 proposes the experimental validation and discussion of the presented method. More specifically, Section 2.1 establishes the trajectory equation for the cutting edge in LTUVAM, enabling the formula derivation of the tool’s axial velocity and angular velocity in Section 3.1; these derived formulas are utilized in the finite element simulation to simulate the variations in the tool’s motion caused by LTUVAM. Section 2.2 divides the surface formation during the CF/PPS composite milling process into four stages; when combined with the finite element modeling of the UD-CF/PPS composites machining process proposed in Section 3.2, it forms the foundation for the material removal and machined surface formation in the finite element analyses. Such finite element analyses are then utilized in Section 4 to compare with experimental results to verify the effectiveness of the method described in this paper.

## 2. The Mechanism of the Longitudinal Torsional Ultrasonic Vibration-Assisted Milling of CF/PPS Composites

### 2.1. Kinematic Analysis of the Longitudinal Torsional Ultrasonic Vibration-Assisted Milling

**Figure 1:**
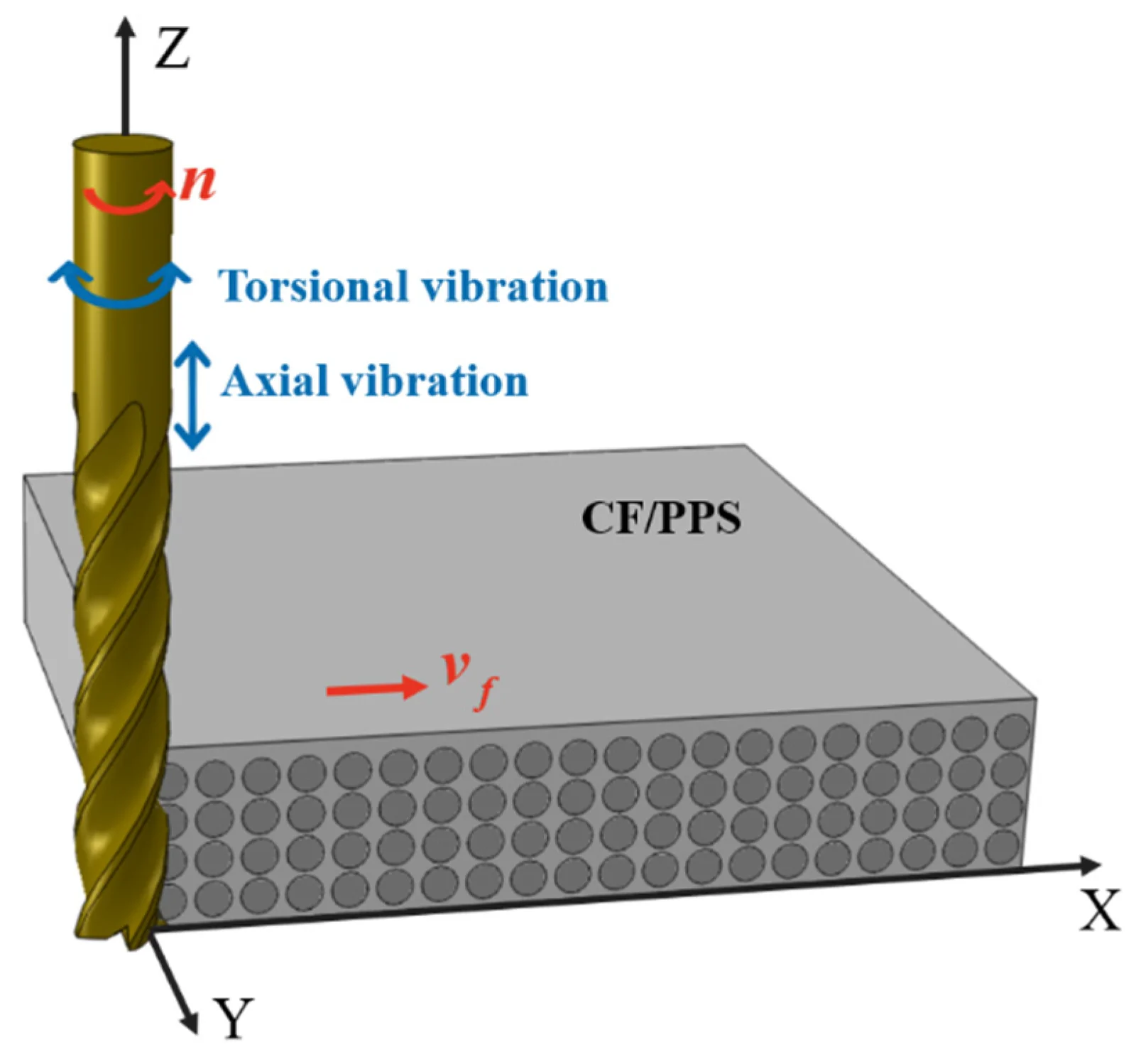
Schematic diagram of a longitudinal torsional ultrasonic vibration-assisted milling process.

During the longitudinal torsional ultrasonic vibration-assisted milling (LTUVAM) process, compared to conventional milling (CM), in addition to its own rotational motion and feed motion, the milling cutter also undergoes axial vibration along the tool axis and torsional vibration in the direction of the spindle’s rotation. A spatial rectangular coordinate system is established with the tool axis as the Z-axis; the ultrasonic-assisted milling process is illustrated in Figure 1. The cutter feeds in the X-axis direction, while the spindle rotates in the X-Y plane. The ultrasonic vibrations perform periodic reciprocating motion along the Z-axis and simultaneously exhibit torsional motion around the tool axis.

For conventional milling process, the motion equation for the tool center can be defined as

(1) $$\left\{\begin{array}{c} x_{0}=V_{f}\cdot t\\ y_{0}=0\\ z_{0}=h \end{array}\right.$$

where $V_{f}$ is the feed speed, *t* is the machining time. Then, the displacement function of the tool’s cutting edge can be derived:

(2) $$\left\{\begin{array}{c} x_{i}(t)=V_{f}t+R\;sin(\frac{2\pi nt}{60})\\ y_{i}(t)=R\;cos(\frac{2\pi nt}{60})\\ z_{i}(t)=0 \end{array}\right.$$

where R is the radius of the cutter, and n is the spindle speed.

In the X-Y plane, the tool’s cutting edge in conventional milling process exhibits two forms of motion: rotation of the milling cutter around the Z-axis and translation along the feed direction. The combination of these two motions forms a trochoidal trajectory for the cutting edge of a milling cutter.

Axial ultrasonic-assisted milling applies axial ultrasonic vibration to the tool in addition to the motions of the conventional milling. Therefore, the trajectory equation for the tool center during axial ultrasonic-assisted milling is

(3) $$\left\{\begin{array}{c} x_{0}=V_{f}\cdot t\\ y_{0}=0\\ z_{0}=A\sin(2\pi ft+\theta_{a0}) \end{array}\right.$$

where A is the axial ultrasonic amplitude, f is the vibration frequency, and $\theta_{a0}$ is the initial phase of the axial ultrasonic vibration. Then, the motion displacement function of the cutting edge can be derived as

(4) $$\left\{\begin{array}{c} x_{0}=V_{f}\cdot t\\ y_{0}=0\\ z_{0}=A\sin(2\pi ft+\theta_{a0}) \end{array}\right.$$

Similarly, torsional ultrasonic-assisted milling generates a periodic angular deviation in the spindle rotation motion of the conventional milling, causing a variation in the actual rotational angle of the tool. The torsional vibration angle of the tool ($\theta_{t}$) is

(5) $$\theta_{t}=\frac{B}{R}\cdot \cos(2\pi ft+\theta_{t0})$$

where B is the torsional amplitude, and $\theta_{t0}$ is the initial phase of the torsional ultrasonic vibration. Superimposing the ultrasonic torsional vibration onto the tool spindle rotation angle, it is feasible to derive the actual torsional angle of the cutting tool ($\theta_{at}$) when torsional ultrasonic vibration is applied:

(6) $$\theta_{at}=\frac{2\pi nt}{60}+\theta_{t}=\frac{2\pi nt}{60}+\frac{B}{R}\cdot \cos(2\pi ft+\theta_{t0})$$

In Figure 1, torsional ultrasonic vibration acts in the X-Y plane, while axial ultrasonic vibration acts in the Z-axis direction. Combining both torsional and axial ultrasonic vibrations, the trajectory equation for the cutting edge in longitudinal torsional ultrasonic vibration-assisted milling is established as

(7) $$\left\{\begin{array}{c} x_{i}(t)=V_{f}t++R\sin(\frac{2\pi nt}{60}+\frac{B}{R}\cos(2\pi ft+\theta_{t0}))\\ y_{i}(t)=R\cos(\frac{2\pi nt}{60}+\frac{B}{R}\cos(2\pi ft+\theta_{t0}))\\ z_{i}(t)=A\sin(2\pi ft+\theta_{a0}) \end{array}\right.$$

**Figure 2:**
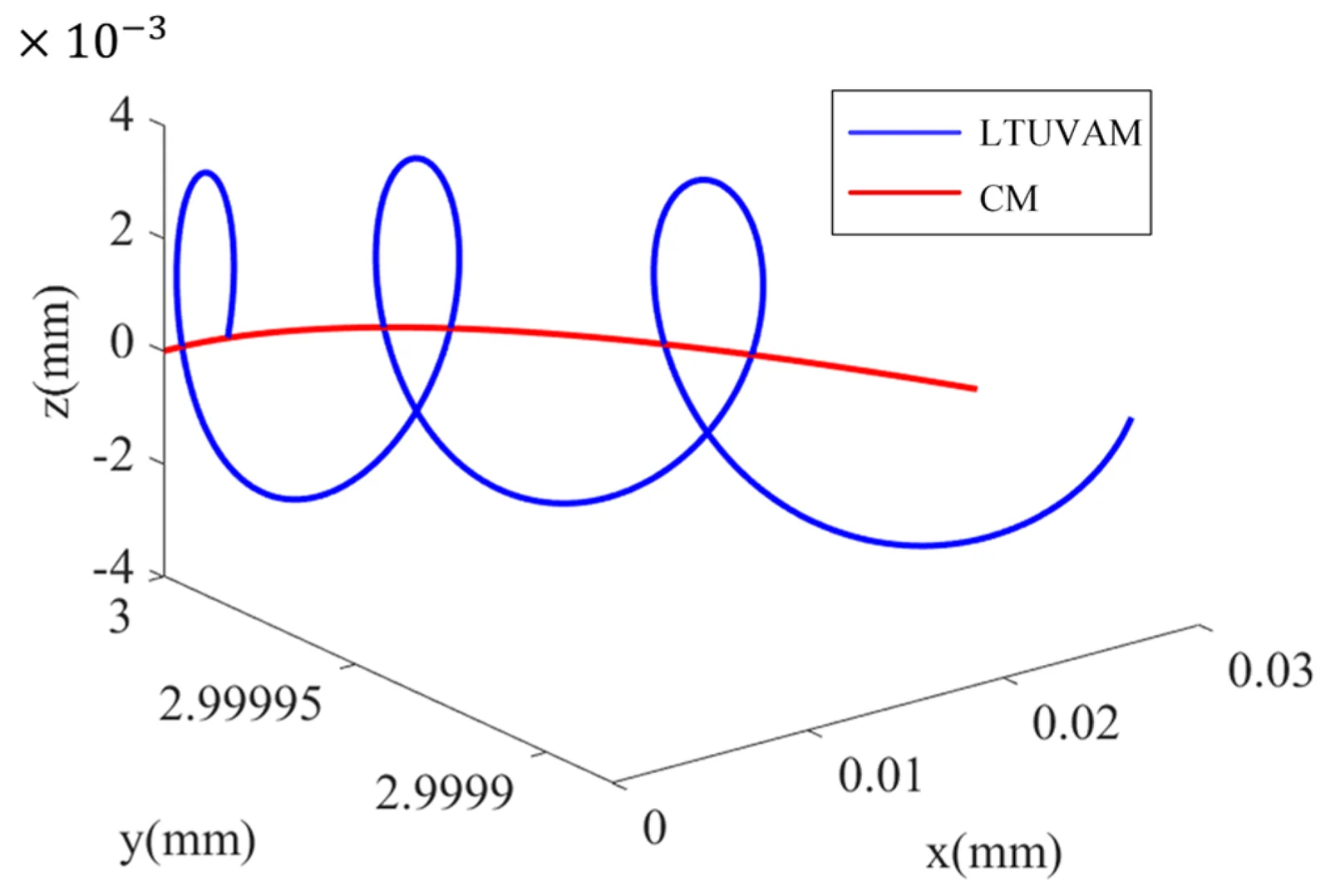
The displacement function profiles for LTUVAM and CM processes.

Based on Equation (7), a comparative illustration of the displacement function profiles for longitudinal torsional ultrasonic vibration-assisted milling (LTUVAM) and conventional milling (CM) is proposed in Figure 2.

### 2.2. The Mechanism of Surface Formation During CF/PPS Milling Process

**Figure 3:**
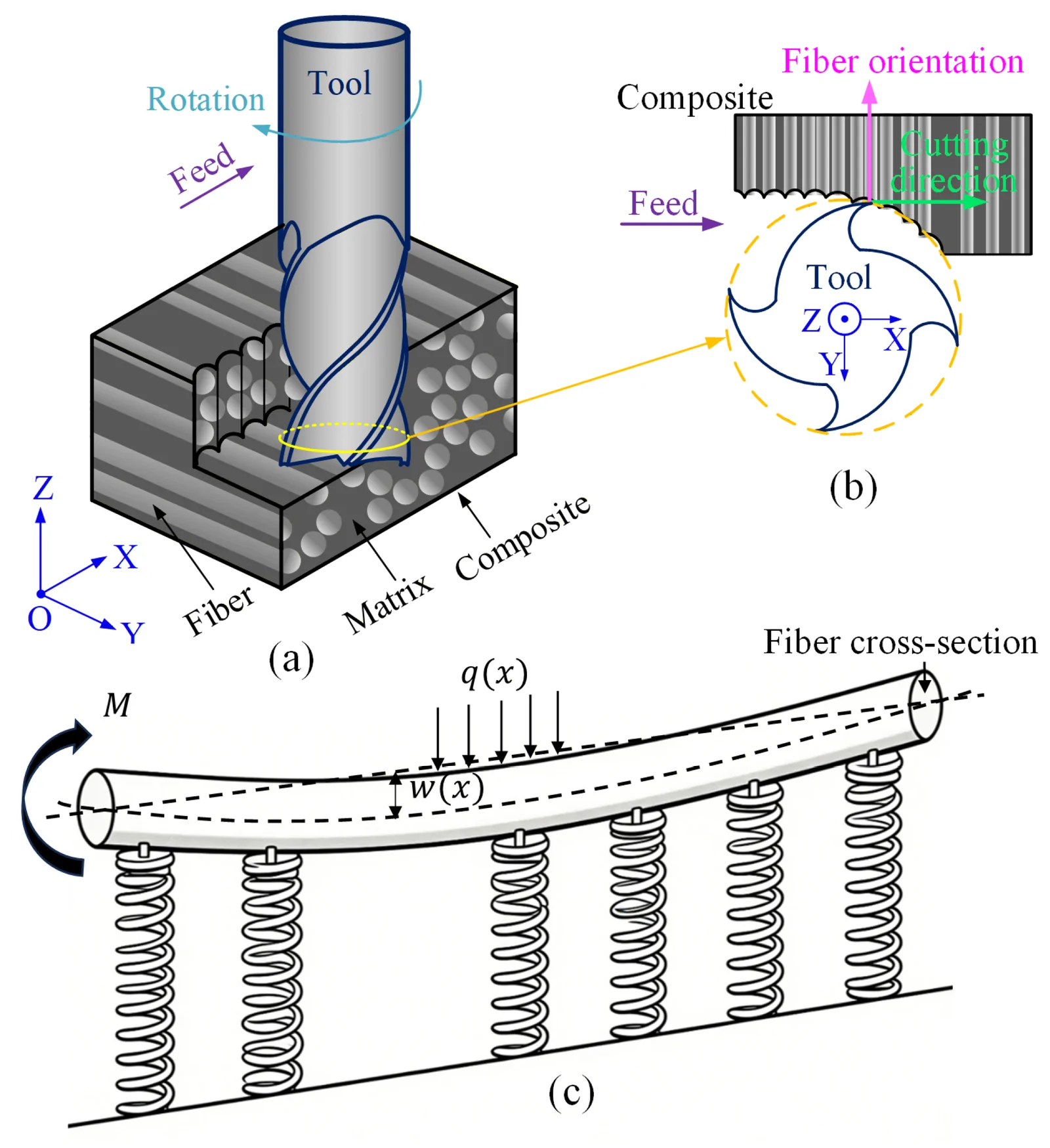
(**a**) A general view and (**b**) a cross-section of an UD-CF/PPS milling process; (**c**) a schematic diagram of the force acting on a fiber.

An unidirectional CF/PPS composite milling process is illustrated in Figure 3a, and a cross-section of cutting area is demonstrated in Figure 3b; it can be observed that the cutting edge begins to cut the chip fibers at an angle of approximately 90°. Therefore, the surface formation during this milling process can be divided into four stages: the extrusion and elastic deformation stage, the fiber crack initiation and brittle fracture stage, the chip formation and matrix plastic flow stage, and the surface finalization and damage evolution stage.

(1) During the extrusion and elastic deformation stage, when the cutting edge cuts perpendicular to the fiber orientation, the fibers are primarily subjected to a combination of transverse shear and bending loads. As the cutting edge approaches the workpiece, it initially exerts a compressive effect on the material. Since the elastic modulus of the PPS matrix is lower than the transverse modulus of the carbon fiber, the matrix undergoes elastic compressive deformation first. This deformation is transmitted to carbon fibers through the interface, causing the carbon fibers to bend under the transverse load. At this stage, the carbon fiber can be simplified as an Euler–Bernoulli beam laid on the PPS matrix, as schematically illustrated in Figure 3c. Based on this model, the bending normal stress in the fiber and the interfacial shear stress can be derived.

Based on the analysis above, the differential equation of the bending deflection curve for fiber can be derived as

(8) $$E_{f}I\frac{d^{4}w}{dx^{4}}+kw=q(x)$$

In Equation (8), w is the fiber bending deflection, I is the moment of inertia of the fiber interface (for a circular cross-section, $I=\pi d_{f}^{4}/64$, where $d_{f}$ is the fiber diameter), and k is the foundation stiffness coefficient (which could be calculated by $k=\pi E_{m}d/\left(2-2\nu \right)$, where $E_{m}$ is the modulus of elasticity for the matrix, d is the fiber diameter, ν is the Poisson ratio of the matrix), and q(x) is the distributed load from the rake face on the fiber.

The maximum bending normal stress in the fiber ($\sigma_{f_{max}}$) is

(9) $$\sigma_{f_{max}}=\frac{M_{max}d_{f}}{2I}$$

where $M_{max}$ is the maximum bending moment of the fiber; when $\sigma_{f_{max}}$ reaches the transverse flexural strength of the fiber, transverse cracks form on the side of the fiber.

The fiber-matrix interfacial shear stress $\tau_{i}(x)$ is

(10) $$\tau_{i}(x)=\frac{dF_{s}}{dx}\cdot \frac{1}{\pi d_{f}}$$

where $F_{s}$ is the shear stress at the fiber cross-section; when $\tau_{i}(x)$ reaches the interfacial shear strength, micro-cracks initiate at the interface.

**Figure 4:**
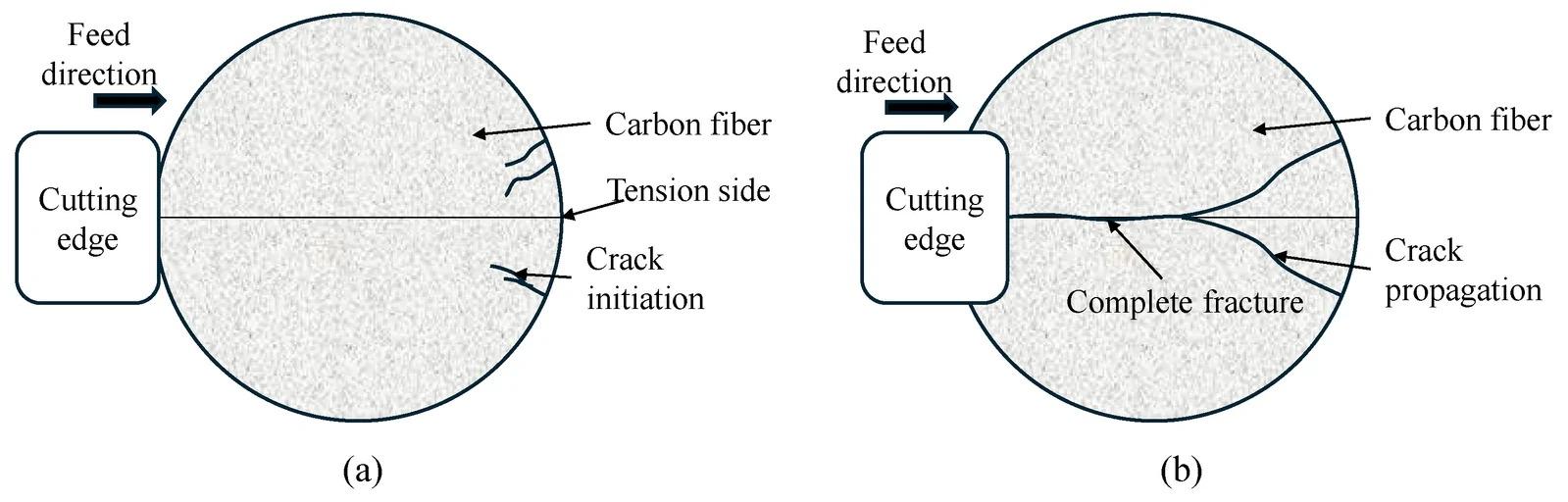
The schematic diagrams of (**a**) the initial fiber crack and (**b**) the fiber fracture.

(2) During the fiber crack initiation and brittle fracture stage, with the feed of the cutting edge, the fiber bending increases and results in significant stress concentration. When the bending normal stress in the fiber reaches its transverse flexural strength, micro-cracks first appear at the stress concentration points on the tensile side of the fiber. These cracks propagate rapidly along the radial direction of the fiber, eventually penetrating the fiber cross-section and causing fiber fracture, as illustrated in Figure 4.

As carbon fiber is a high-stiffness, low-ductility material, it always reaches its fracture limit under relatively small transverse deformation. Under ideal cutting conditions, the fiber breaks within the shear plane on the tool’s rake face, resulting in a clean cut and a smooth surface. However, when cutting forces vary or the cutting edge becomes blunted, fibers are prone to subsurface fracture beneath the machined surface; the root of a fractured fiber tends to experience interfacial debonding during subsequent machining, which is the primary source of fiber pull-out and surface pitting defects.

(3) During the chip formation and matrix plastic flow stage, after the fiber completely fractures, the fractured fiber segments and the surrounding PPS matrix together form chips, which flow out along the tool’s rake face. The heat generated by friction between the tool’s rake face and the chips, coupled with the heat generated by material deformation, causes the temperature in the cutting area to rise rapidly. When the temperature exceeds the glass transition temperature of PPS, the matrix softens and its viscosity drops significantly. When the temperature exceeds the melting point, the matrix experiences localized melting. Meanwhile, fibers that have not been completely cut are pulled during the flow of chips, causing interfacial cracks to spread along the fiber axis and resulting in extensive interfacial debonding.
(4) During the surface finalization and damage evolution stage, after the cutting edge leaves the machining area, the cutting force decreases to zero, causing the compressed PPS matrix to experience a partial elastic rebound. However, carbon fibers that have experienced brittle fracture lack the ability to recover from deformation, resulting in a microscopic height difference between the fiber and the matrix. Simultaneously, the molten PPS matrix rapidly cools and recrystallizes in the air, ultimately solidifying to form the machined surface. At this stage, subsurface damage ultimately reaches its final form.

## 3. Finite Element Modeling of the Longitudinal Torsional Ultrasonic Vibration-Assisted Milling of UD-CF/PPS Composites

### 3.1. A Finite Element Simulation Method for LTUVAM

To model ultrasonic vibrations in finite element simulations, a method based on periodic curves is proposed; by employing the Fourier formula, the amplitude of periodic ultrasonic vibrations (Amp) can be described as follows:

(11) $$Amp=A_{0}+\sum_{n=1}^{m}\left[A_{n}cos\;n\omega \left(t-t_{0}\right)+B_{n}sin\;n\omega \left(t-t_{0}\right)\right]$$

where $A_{0}$ is the initial amplitude, m is the number of the Fourier Series, and ω is the vibration frequency.

Since LTUVAM couples longitudinal vibration (along the axial direction) and torsional vibration (rotation along the axial direction), it generates a complex three-dimensional vibration motion. This motion directly affects the tool’s axial velocity and angular velocity; therefore, by simultaneously varying the tool’s axial velocity and angular velocity, it is feasible to simulate the variations in tool’s motion caused by LTUVAM.

First, assuming an initial phase of 0 and applying axial vibration to the tool according to Equation (3), since Equation (3) is the trajectory equation for the tool, differentiated processing of Equation (3) could provide the equation for the tool’s axial velocity:

(12) $$V_{Z}=2\pi fA\cos(2\pi ft)$$

Similarly, differentiating Equation (6) could produce the tool’s torsional angular velocity:

(13) $$\omega =2\pi n/60+2\pi f\frac{B}{R}\sin(2\pi ft)$$

By separately integrating Equations (12) and (13), the amplitudes of the tool’s axial displacement and torsional angle at any instance could be obtained, enabling the modeling of the tool’s longitudinal and torsional vibrations.

### 3.2. Finite Element Modeling of the UD-CF/PPS Composites Machining Process

**Figure 5:**
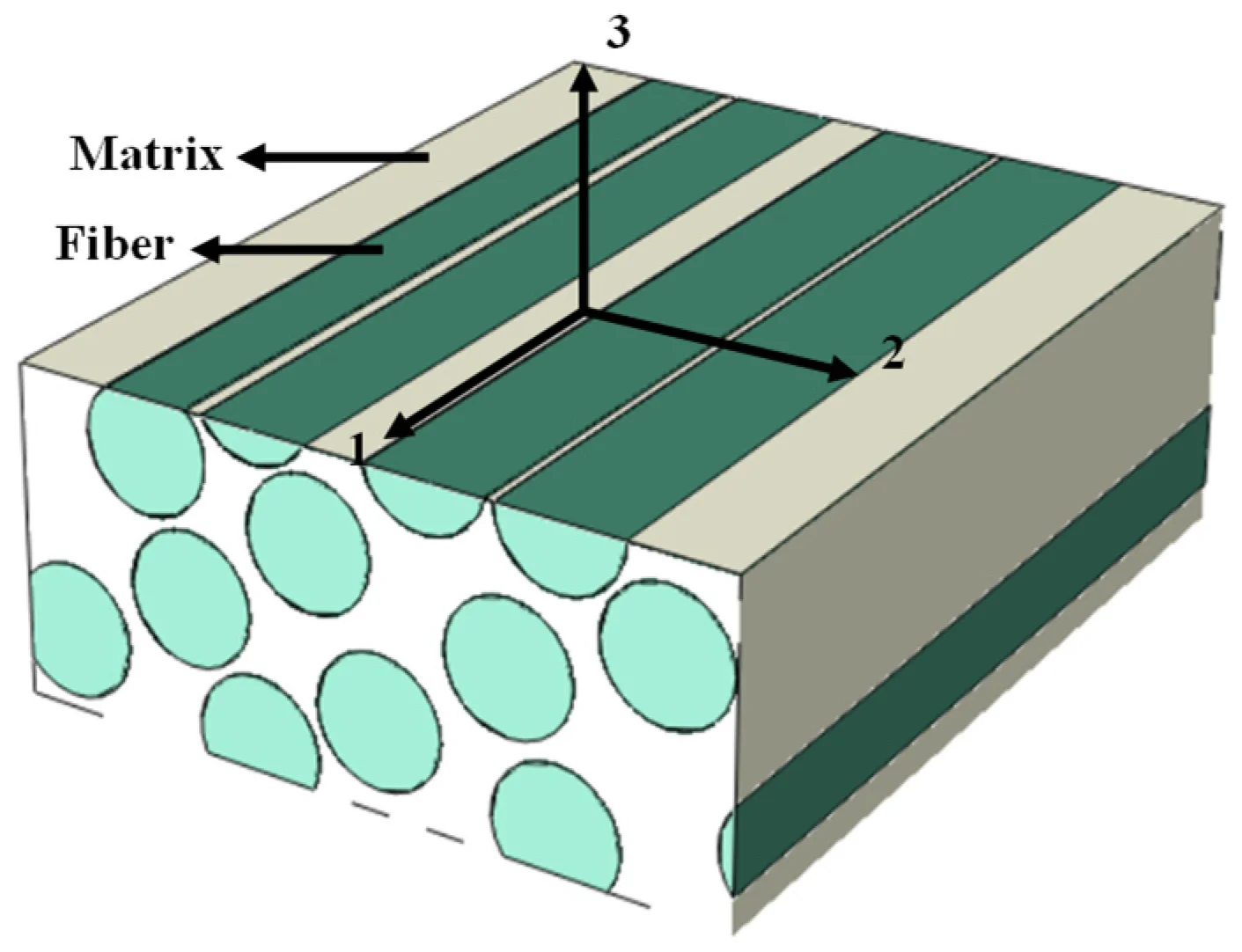
The definitions of the fiber orientation and directions.

Since UD-CF/PPS composites feature anisotropic mechanical properties, a progressive damage criterion based on the Hashin model is employed to characterize the damage behavior of fibers and the matrix. The definitions of the fiber orientation and directions are presented in Figure 5, where the direction 1 is the fiber orientation, direction 2 is perpendicular to the fiber orientation, and direction 3 is perpendicular to both direction 1 and direction 2.

The damage modes of the UD-CF/PPS composites can be classified into the following four types:

(1) The fiber tension mode (FT):
  (14) $$F_{ft}={\left(\frac{\sigma_{11}}{X_{T}}\right)}^{2}+\alpha \left(\frac{\sigma_{12}^{2}+\sigma_{13}^{2}}{S_{12}^{2}}\right)\geq 1$$
(2) The fiber compression mode (FC):
  (15) $$F_{fc}={\left(\frac{\left|\sigma_{11}\right|}{X_{C}}\right)}^{2}\geq 1$$
(3) The matrix tension mode (MT):
  (16) $$F_{mt}={\left(\frac{\sigma_{22}+\sigma_{33}}{Y_{T}}\right)}^{2}+\frac{1}{S_{23}^{2}}(\sigma_{23}^{2}-\sigma_{22}\sigma_{33})+\left(\frac{\sigma_{12}^{2}+\sigma_{13}^{2}}{S_{12}^{2}}\right)\geq 1$$
(4) The matrix compression mode (MC):
  (17) $$F_{mc}=\left[{\left(\frac{Y_{C}}{2S_{23}}\right)}^{2}-1\right]\frac{\sigma_{22}+\sigma_{33}}{Y_{C}}+{\left(\frac{\sigma_{22}+\sigma_{33}}{2S_{23}}\right)}^{2}+\frac{1}{S_{12}^{2}}(\sigma_{12}^{2}+\sigma_{13}^{2})+\frac{1}{S_{23}^{2}}(\sigma_{23}^{2}-\sigma_{22}\sigma_{33})\geq 1$$
  where $X_{C}$ and $X_{T}$ are longitudinal tensile and compressive strengths along the fiber orientation, respectively. $Y_{T}$ and $Y_{C}$ separately represent the transverse tensile and compressive strengths. $S_{12}$, $S_{13}$ and $S_{23}$ are the shear strengths in three orthogonal directions.

When a UD-CF/PPS composite material is subjected to external loads during processing, once it reaches the damage initiation criterion, the corresponding damage variable (with a range of [0, 1]) will begin to evolve based on a fracture energy criterion to avoid mesh dependency. To perform energy regularization, the stress and strain states need to be converted into scalar variables for each damage mode. The equivalent strain $\varepsilon_{eq}^{mode}$ corresponding to the four damage modes above can be first calculated based on the current strain and stress, then the equivalent displacement ($\delta_{eq}^{mode}$) can be derived as

(18) $$\delta_{eq}^{mode}=L_{c}\cdot \varepsilon_{eq}^{mode}$$

where $L_{c}$ is the characteristic length of the element; the equivalent strain calculations for different damage modes are as follows:

(a) The fiber tension mode ($\sigma_{11}\geq 0$):
  (19) $$\varepsilon_{eq}^{ft}=\sqrt{\left\langle \varepsilon_{11}\rangle^{2}+k(\varepsilon_{12}^{2}+\varepsilon_{13}^{2}\right)}$$
  (20) $$\sigma_{eq}^{ft}=\frac{L_{c}}{\delta_{eq}^{ft}}\left(\left\langle \sigma_{11}\rangle \langle \varepsilon_{11}\rangle +k(\sigma_{12}\varepsilon_{12}+\sigma_{13}\varepsilon_{13}\right)\right)$$
(b) The fiber compression mode ($\sigma_{11}<0$):
  (21) $$\varepsilon_{eq}^{fc}=\langle -\varepsilon_{11}\rangle$$
  (22) $$\sigma_{eq}^{fc}=\frac{L_{c}}{\delta_{eq}^{fc}}\left(\left\langle -\sigma_{11}\rangle \langle -\varepsilon_{11}\right\rangle \right)$$
(c) The matrix tension mode ($\sigma_{22}+\sigma_{33}\geq 0$):
  (23) $$\varepsilon_{eq,22}^{mt}=\sqrt{\left\langle \varepsilon_{22}\rangle^{2}+k(\varepsilon_{12}^{2}+\varepsilon_{23}^{2}\right)}$$
  (24) $$\sigma_{eq,22}^{mt}=\frac{L_{c}}{\delta_{eq,22}^{mt}}\left(\left\langle \sigma_{22}\rangle \langle \varepsilon_{22}\rangle +k(\sigma_{12}\varepsilon_{12}+\sigma_{23}\varepsilon_{23}\right)\right)$$
  (25) $$\varepsilon_{eq,33}^{mt}=\sqrt{\left\langle \varepsilon_{33}\rangle^{2}+k(\varepsilon_{13}^{2}+\varepsilon_{23}^{2}\right)}$$
  (26) $$\sigma_{eq,33}^{mt}=\frac{L_{c}}{\delta_{eq,33}^{mt}}\left(\left\langle \sigma_{33}\rangle \langle \varepsilon_{33}\rangle +k(\sigma_{13}\varepsilon_{13}+\sigma_{23}\varepsilon_{23}\right)\right)$$
(d) The matrix compression mode ($\sigma_{22}+\sigma_{33}<0$):
  (27) $$\varepsilon_{eq,22}^{mc}=\langle -\varepsilon_{22}\rangle$$
  (28) $$\sigma_{eq,22}^{mc}=\frac{L_{c}}{\delta_{eq,22}^{mc}}(\langle -\sigma_{22}\rangle \langle -\varepsilon_{22}\rangle )$$
  (29) $$\varepsilon_{eq,33}^{mc}=\langle -\varepsilon_{33}\rangle$$
  (30) $$\sigma_{eq,33}^{mc}=\frac{L_{c}}{\delta_{eq,33}^{mc}}\left(\left\langle -\sigma_{33}\rangle \langle -\varepsilon_{33}\right\rangle \right)$$
  where ⟨⟩ is the Macaulay bracket operator; the calculation is $\left\langle x\right\rangle =(x+|x|/2)$.

After obtaining the current equivalent displacement $\delta_{eq}$, the damage variable *d* changes accordingly, and it can be calculated as

(31) $$d_{I}=\frac{\delta_{I,eq}^{f}\left(\delta_{I,eq}-\delta_{I,eq}^{0}\right)}{\delta_{I,eq}(\delta_{I,eq}^{f}-\delta_{I,eq}^{0})}\;(d_{I}\in [0\;1],\;I=ft,fc,mt,mc)$$

where $\delta_{I,eq}^{0}$ is the equivalent displacement at the initiation of damage, and $\delta_{I,eq}^{f}$ is the equivalent displacement at damage failure. The calculations of these variables are

(32) $$\delta_{I,eq}^{0}=\delta_{I,eq}/\sqrt{F_{I}}$$

(33) $$\delta_{I,eq}^{f}=2G_{I}/(\sigma_{I,eq}/\sqrt{F_{I}})$$

where $\sigma_{I,eq}$ is the equivalent stress corresponding to the damage mode I.

When the stress reaches the damage threshold, the material does not fracture immediately but retains some load-bearing capacity. During this damage evolution process, the material’s stiffness exhibits a progressively diminishing characteristic, and its ability to resist external loads gradually decreases. To accurately characterize the stiffness reduction behavior of UD-CF/PPS composite materials, additional damage variables need to be incorporated. The compliance matrix of the material after damage ($S_{d}$) is expressed as

(34) $$S_{d}=\left[\begin{array}{cccccc} \frac{1}{d_{f}E_{11}} & -\frac{\nu_{21}}{E_{22}} & -\frac{\nu_{31}}{E_{33}} & 0 & 0 & 0\\ -\frac{\nu_{12}}{E_{11}} & \frac{1}{d_{m}E_{22}} & -\frac{\nu_{32}}{E_{33}} & 0 & 0 & 0\\ -\frac{\nu_{13}}{E_{11}} & -\frac{\nu_{23}}{E_{22}} & \frac{1}{E_{33}} & 0 & 0 & 0\\ 0 & 0 & 0 & \frac{1}{d_{f}d_{m}G_{12}} & 0 & 0\\ 0 & 0 & 0 & 0 & \frac{1}{d_{f}d_{m}G_{23}} & 0\\ 0 & 0 & 0 & 0 & 0 & \frac{1}{d_{f}d_{m}G_{31}} \end{array}\right]$$

where $d_{f}$ and $d_{m}$ represent the fiber damage variable and the matrix damage variable, respectively. The stiffness matrix of the material after damage ($C_{d}$) is the inverse of the compliance matrix:

(35) $$\begin{array}{c} C_{d}=\\ \frac{1}{\Delta }\left[\begin{array}{cccccc} d_{f}E_{11}\left(1-d_{m}\nu_{23}\nu_{32}\right) & d_{f}d_{m}E_{11}\left(\nu_{21}+\nu_{23}\nu_{31}\right) & d_{f}E_{11}\left(\nu_{31}+d_{m}\nu_{21}\nu_{32}\right) & 0 & 0 & 0\\ 0 & d_{m}E_{22}\left(1-d_{f}\nu_{13}\nu_{31}\right) & d_{m}E_{22}\left(\nu_{32}+d_{f}\nu_{12}\nu_{31}\right) & 0 & 0 & 0\\ 0 & 0 & E_{33}\left(1-d_{f}d_{m}\nu_{12}\nu_{21}\right) & 0 & 0 & 0\\ 0 & 0 & 0 & \Delta d_{f}d_{m}G_{12} & 0 & 0\\ 0 & 0 & 0 & 0 & \Delta d_{f}d_{m}G_{23} & 0\\ 0 & 0 & 0 & 0 & 0 & \Delta d_{f}d_{m}G_{13} \end{array}\right] \end{array}$$

where

(36) $$\left\{\begin{array}{c} d_{f}=(1-d_{ft})(1-d_{fc})=\max\left({\left(1-S_{mt}d_{mt}\right)}^{2},{\left(1-S_{mc}d_{mc}\right)}^{2}\right)\\ d_{m}=\max\left({\left(1-S_{mt}d_{mt}\right)}^{2},{\left(1-S_{mc}d_{mc}\right)}^{2}\right)\\ \Delta =1-d_{f}d_{m}\nu_{12}\nu_{21}-d_{m}\nu_{23}\nu_{32}-d_{f}\nu_{13}\nu_{31}-2d_{f}d_{m}\nu_{21}\nu_{32}\nu_{13} \end{array}\right.$$

In Equation (36), $d_{ft}$, $d_{fc}$, $d_{mt}$ and $d_{mc}$ are damage variables of fibers and the matrix under tensile and compressive loads, respectively.

During the simulation, the finite element model includes carbon fibers, the PPS matrix, and the interfacial layer. At the microscopic level, carbon fibers are defined as orthotropic, linearly elastic materials. The maximum stress criterion is adopted as the damage initiation threshold; when the stress in any direction of the fiber reaches its strength limit, the fiber element is deemed to experience failure. At the macroscopic scale, the Hashin progressive damage criterion is embedded in the Abaqus 2023 VUMAT subroutine, which includes four damage modes: the fiber tension mode, the fiber compression mode, the matrix tension mode and the matrix compression mode.

For the PPS matrix, the Johnson–Cook damage model is adopted. The damage initiation threshold is determined by the Johnson–Cook failure strain formula, taking into account the coupled effects of stress triaxiality, strain rate, and temperature. The damage initiation is deemed to occur when the cumulative equivalent plastic strain reaches the critical failure strain, and the damage variable continues to increase with plastic deformation, causing the stiffness to decline progressively until the element fails completely.

A cohesive model is adopted for the fiber–matrix interface, with the quadratic nominal stress criterion utilized as the damage initiation threshold. When the normalized quadratic sum of the normal tensile force and the two tangential tensile forces reaches 1, damage initiates at the interface. The damage evolution follows the Benzeggagh–Kenane (BK) criterion, and the stiffness progressively degrades until the interface completely loses its load-bearing capacity.

To bond fibers and the matrix in finite element modeling, at the microscopic scale, zero-thickness cohesive elements are inserted between the fiber phase and the matrix phase. The bonding and debonding behavior of the two phases is characterized by using the traction–separation constitutive relationship of the cohesive elements, enabling accurate simulation of the initiation and propagation of interfacial cracks. At the macroscopic scale, the characteristics of equivalent orthotropic materials are obtained through the homogenization of representative volume elements (RVE), which are then utilized to characterize the mechanical behavior of fiber-reinforced materials, and a continuum damage model is employed to describe the progressive damage.

## 4. Experimental Validation and Discussion of CF/PPS Composites Milling Results

In the following, the experimental and simulated forces during LTUVAM and CM processes are presented to verify the proposed model, and the relevant machined surfaces are then provided to demonstrate the effectiveness of LTUVAM on machining quality improvement. Various cutting tools are also presented to further verify the effectiveness of LTUVAM and to assess how differences in cutting tools affect the CF/PPS composites machining results.

### 4.1. Experimental Setup and Measurement of Longitudinal Torsional Ultrasonic Parameters

The workpieces utilized in experiments were carbon fiber-reinforced polyphenylene sulfide (CF/PPS) unidirectional laminates, with polyphenylene sulfide resin as the matrix and T700 carbon fiber (JIE DIAN NEW MATERIAL, Weifang, China) as the reinforcement phase. The carbon fiber diameter is approximately 7 μm, the fiber volume fraction is 60%, and the workpiece dimension is 110 mm × 90 mm × 4 mm. The relevant milling trials were conducted using a five-axis milling machine (KMC400S U), together with three different cutters and a three-component dynamometer (9257B, Kistler, Winterthur, Switzerland). Cutting forces acting on the workpiece in x and y directions were measured, and the feed direction was parallel to x direction.

**Figure 6:**
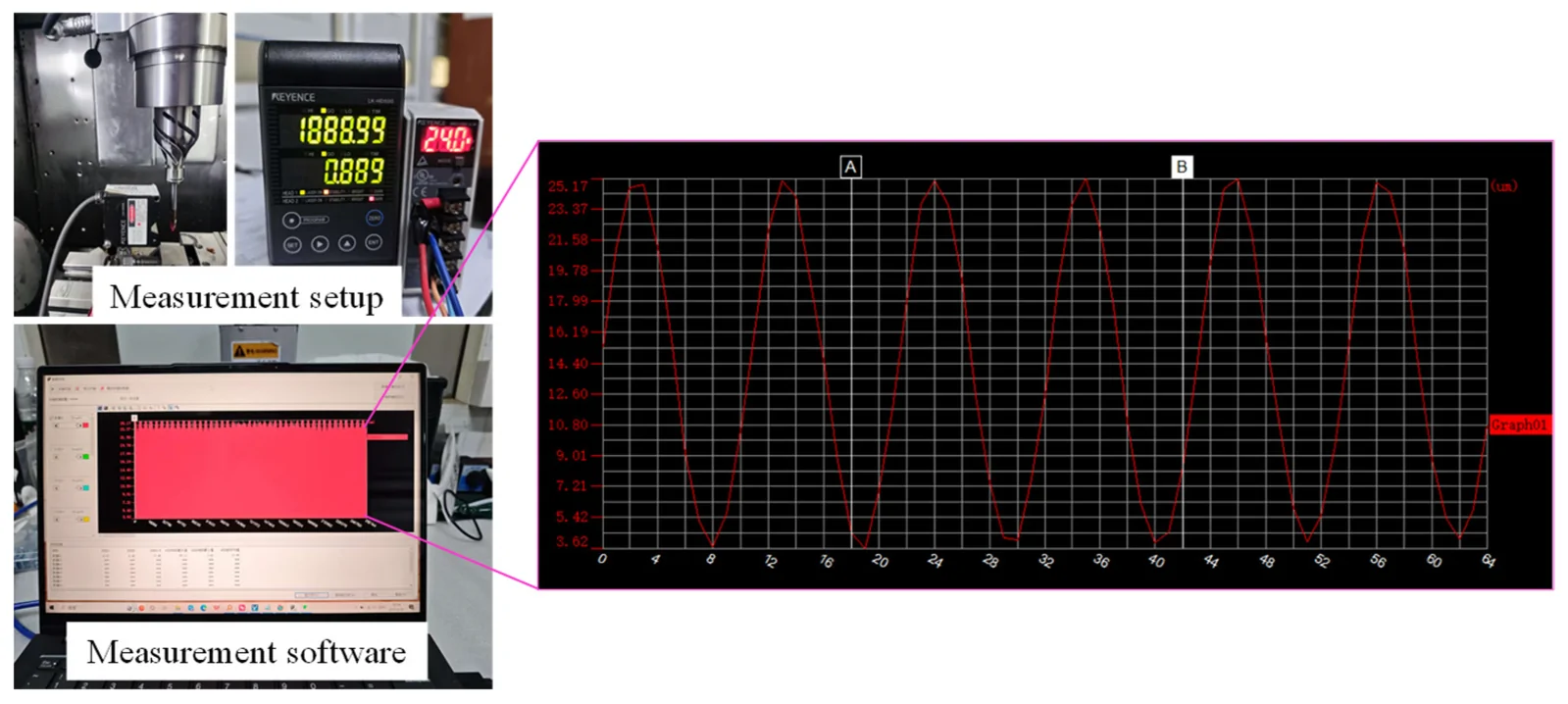
The measurement setup of longitudinal torsional ultrasonic parameters.

To measure the longitudinal torsional ultrasonic parameters during LTUVAM process, a LK-G5000 series high-speed, high-precision laser displacement sensor (KEYENCE CORPORATION, Itasca, IL, USA) was utilized to calibrate the tool tip amplitude. The linearity of this sensor is 1.2 μm, and the repeatability of this sensor is 0.02 μm. To ensure measurement accuracy, multiple repeat measurements were conducted for each set of cutting-edge amplitude data, and the average value was calculated to minimize random measurement errors. The measurement setup is presented in Figure 6, and the measured longitudinal torsional ultrasonic parameters are proposed in Table 1.

### 4.2. Validation of the Proposed Finite Element Simulation Method in CF/PPS Milling Process

To validate the provided simulation method in CF/PPS milling process, a series of milling trials were conducted under different feed speeds and spindle speeds, with the constant axial depth of cut 4 mm and radial depth of cut 1 mm. The workpiece size is 20 × 20 × 4 mm, fiber volume fraction is 60%, and fiber diameter is 7 μm.

During the simulation, the tool was modeled as a rigid body, and a moving load was applied to the cutting edge. To apply the moving load of the vibration-assisted milling, the basic cutting motions which consisted of spindle rotation and feed motion were first applied, then the longitudinal vibration and torsional vibration were added based on the Abaqus’ periodic amplitude function (in the form of a Fourier series, as presented in Section 3.1). For the workpiece boundary conditions, applying complete fixed constraints to the bottom surface and the side surfaces of the workpiece, which are far from the cutting zone, restricting all translational and rotational degrees of freedom, is consistent with the actual clamping method utilized for the workpiece in the experiment.

Regarding the mesh division in the simulation, for the macroscopic model, an 8-node linear hexahedral reduced integration element (C3D8R) was utilized to perform structured hexahedral meshing, locally refining the mesh in the cutting contact area. For the microscopic model, the carbon fiber and PPS matrix were meshed utilizing 4-node linear tetrahedral elements (C3D4), while the interface layer was modeled utilizing three-dimensional cohesive elements (COH3D8), and the element size in the cutting zone was refined. During the modeling process, sensitivity analysis was performed on the element sizes in the cutting region, and the mesh was progressively refined until key simulation results (such as cutting forces and stress distributions) demonstrated no significant changes with further mesh refinement.

**Figure 7:**
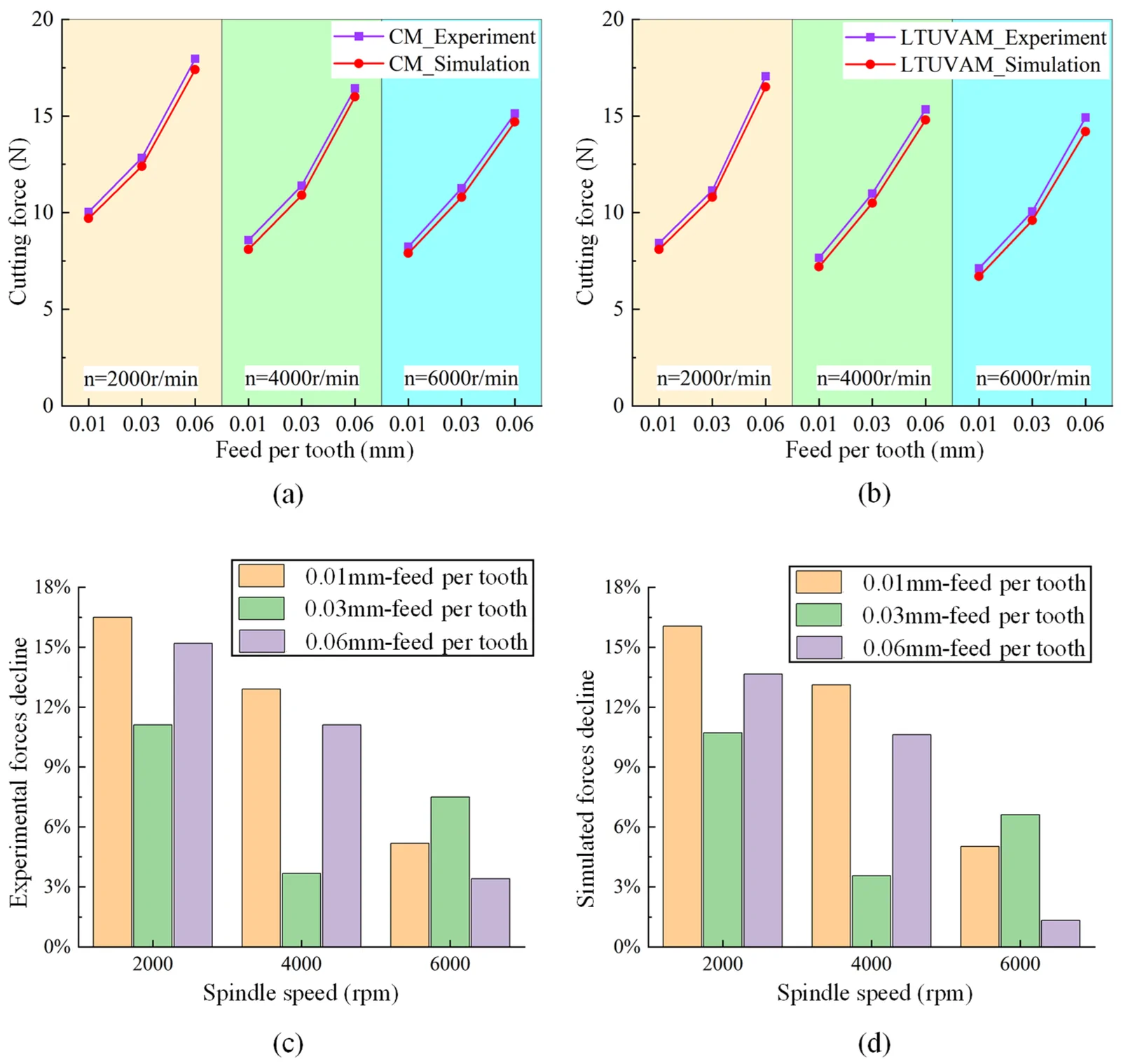
The comparisons of experimental and simulated forces in (**a**) CM and (**b**) LTUVAM processes, the reduction ratios of cutting forces in LTUVAM processes compared to CM processes during (**c**) experiment and (**d**) simulation procedures.

A comparison of experimental cutting forces and simulations from conventional milling (CM) processes utilizing a 6 mm diameter helical cutter is presented in Figure 7a, and a comparison of experimental and simulated cutting forces from longitudinal torsional ultrasonic vibration-assisted milling (LTUVAM) processes is proposed in Figure 7b.

In Figure 7a,b, average values of cutting forces under different machining conditions are provided; it can be observed that the simulated forces show significant agreement with experimental data in both CM and LTUVAM processes. The comparison errors between the experimental and simulated cutting forces are less than 7%. The influencing factors of these errors may come from the following aspects:

(1) Workpiece thickness tolerance: the workpiece was manufactured to a thickness of 4 mm, and this thickness value was also utilized in the simulation. However, the authors conducted the experiment utilizing three workpieces; each workpiece had a slight deviation (less than 1%) from the target thickness. This could affect the axial depth of cut and thus influence the cutting forces.
(2) Fiber diameter tolerance: all fibers were assumed to have a perfect diameter of 7 mm in the simulation, which introduced a certain deviation from the actual material.
(3) Fiber volume fraction tolerance: the workpieces were manufactured with a fiber volume fraction of 60%; the simulation utilized such volume fraction values, which introduced a certain deviation from the actual composite materials.
(4) Modeling error.

To minimize the impact of errors, each set of machining parameters was conducted three times, and the average of the three sets of measurement values was taken as the experimental data corresponding to the specific cutting condition.

Furthermore, compared to CM, cutting forces are noticeably reduced under LTUVAM conditions. To quantify this decline, Figure 7c demonstrates the reduction ratio of experimental cutting forces in LTUVAM processes compared to CM processes, and Figure 7d illustrates the reduction ratio of simulated cutting forces under LTUVAM conditions compared to CM conditions. It can be noticed that in both experiment and simulation results, LTUVAM is more effective at reducing cutting forces under lower spindle speeds. Also, the simulation results not only match the experimental results in average cutting forces but also indicate good agreement with experimental results regarding the cutting force reductions caused by LTUVAM.

**Figure 8:**
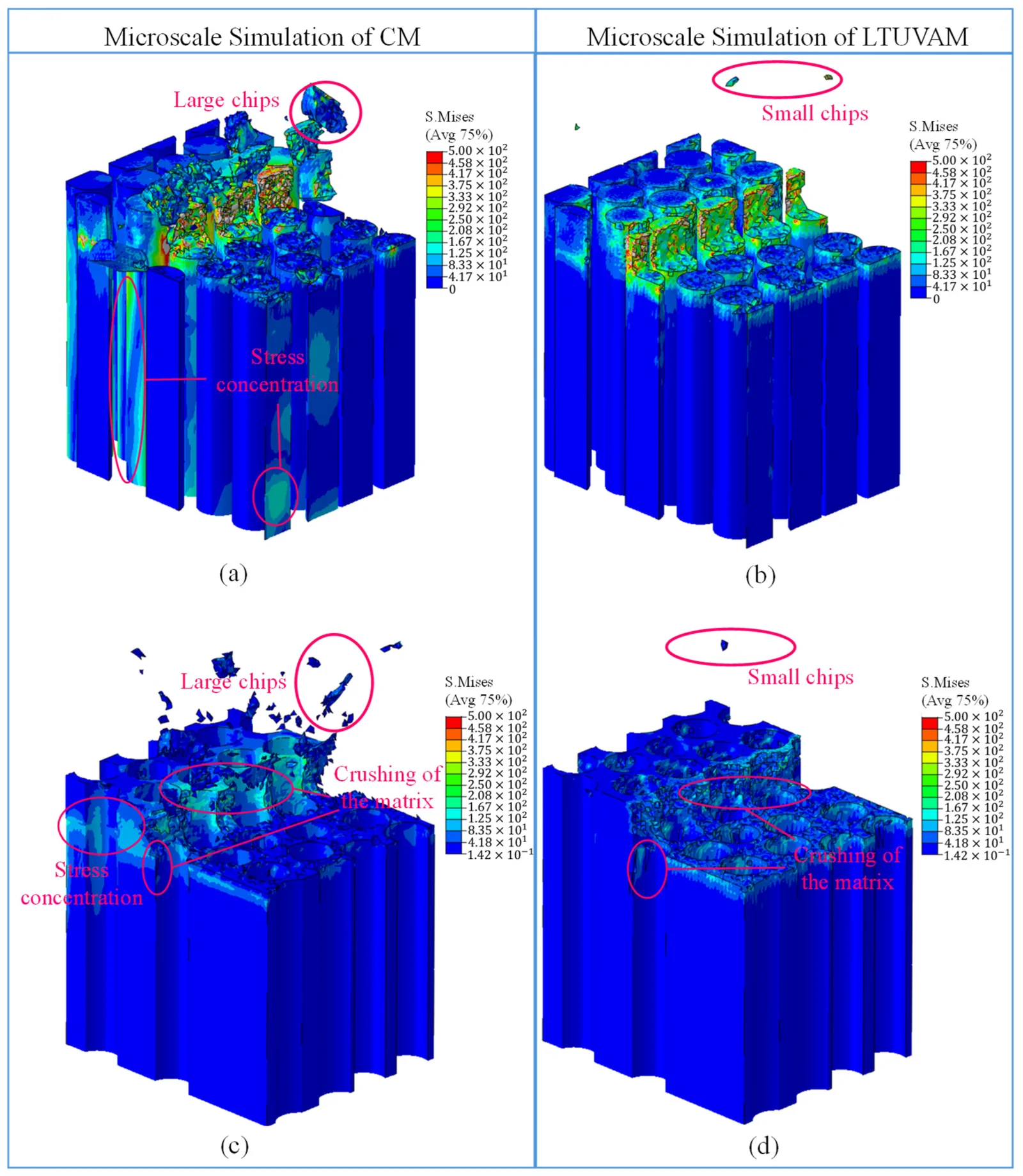
The simulated microscopic stress–strain contours of the fiber cutting process in (**a**) CM and (**b**) LTUVAM procedures, the microscopic stress–strain contours of the matrix cutting process in (**c**) CM and (**d**) LTUVAM conditions.

To further demonstrate the effects of LTUVAM on machined surface topography, Figure 8a presents the microscopic stress–strain contour of the fiber cutting process in CM, and Figure 8b proposes the microscopic stress–strain contour of the fiber cutting process in LTUVAM. It can be observed that CM operation produces larger chips than LTUVAM. Furthermore, during the CM process, the stress field exhibits a diffusive pattern; high-stress regions are not only concentrated at the interface between the cutting edge and the material but also extend along the fiber direction into the interior of the material and beneath the machined surface. This indicates that mechanical loads during the CM process have a propagating effect on the deeper layers of the material, which can potentially induce subsurface damage and bonding failure at the interface. In contrast, under the LTUVAM condition, high-stress areas are effectively limited to a small region near the interface between the cutting edge and the material; the stress response beneath the machined surface is significantly reduced, indicating that the high-frequency pulses of ultrasonic vibration alter the transmission path of the cutting force, concentrating energy more in the chip formation area and reducing disturbance to the material.

Figure 8c demonstrates the microscopic stress–strain contour of the matrix cutting process in CM, indicating that the high-stress region extends forward along the cutting direction, causing extensive stress disturbances in the matrix. Figure 8d provides the microscopic stress–strain contour of the matrix cutting process in LTUVAM, illustrating that high-stress regions are effectively limited near the interface between the cutting edge and the material, and the propagation of stress into the matrix is significantly reduced, resulting in a more localized stress distribution. This also implies that the high-frequency pulses of ultrasonic vibration alter the transmission path of cutting forces within the matrix, concentrating mechanical energy more intensely in the chip-forming region and thus reducing the impact of cutting forces on the matrix.

**Figure 9:**
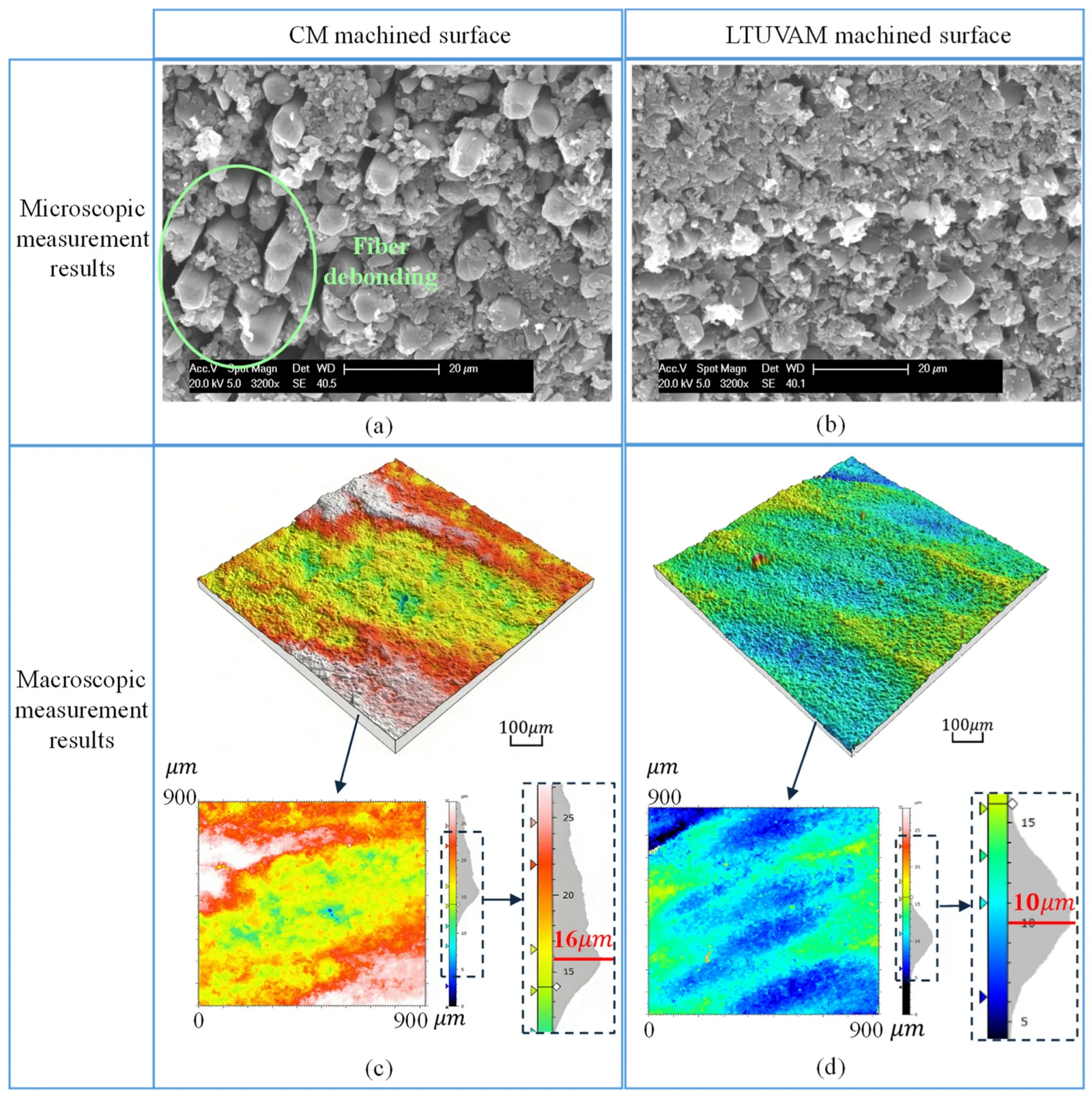
The microscopic topographies of machined surfaces corresponding to (**a**) CM and (**b**) LTUVAM processes; the macroscopic measurements for (**c**) CM and (**d**) LTUVAM machined surfaces.

An example of the machined surface measurements (see Figure 9) also verifies the effectiveness of LTUVAM in improving surface machining quality; both machining operations were under 2000 rpm spindle speed, 0.06 mm feed per tooth, 4 mm axial depth of cut and 1 mm radial depth of cut. Figure 9a,b demonstrates the microscopic topographies of machined surfaces corresponding to CM and LTUVAM operations, respectively. Figure 9c,d separately proposes the macroscopic surface measurements for CM and LTUVAM. It can be observed that both micro- and macro-measurements have affirmed that LTUVAM produces superior surface quality compared to CM. The phenomenon of fiber debonding at the microscopic level could be eliminated when LTUVAM is utilized; at the macro level, the height of machined surfaces could be significantly reduced through LTUVAM.

### 4.3. Validation of the LTUVAM Effectiveness Across Different Tool Geometries and Materials

A 6 mm diameter tungsten steel straight cutter and a 6 mm diameter PCD straight cutter were employed to further verify the LTUVAM effectiveness; the measured longitudinal torsional ultrasonic parameters corresponding to these two cutters have already been proposed in Table 1 above.

**Figure 10:**
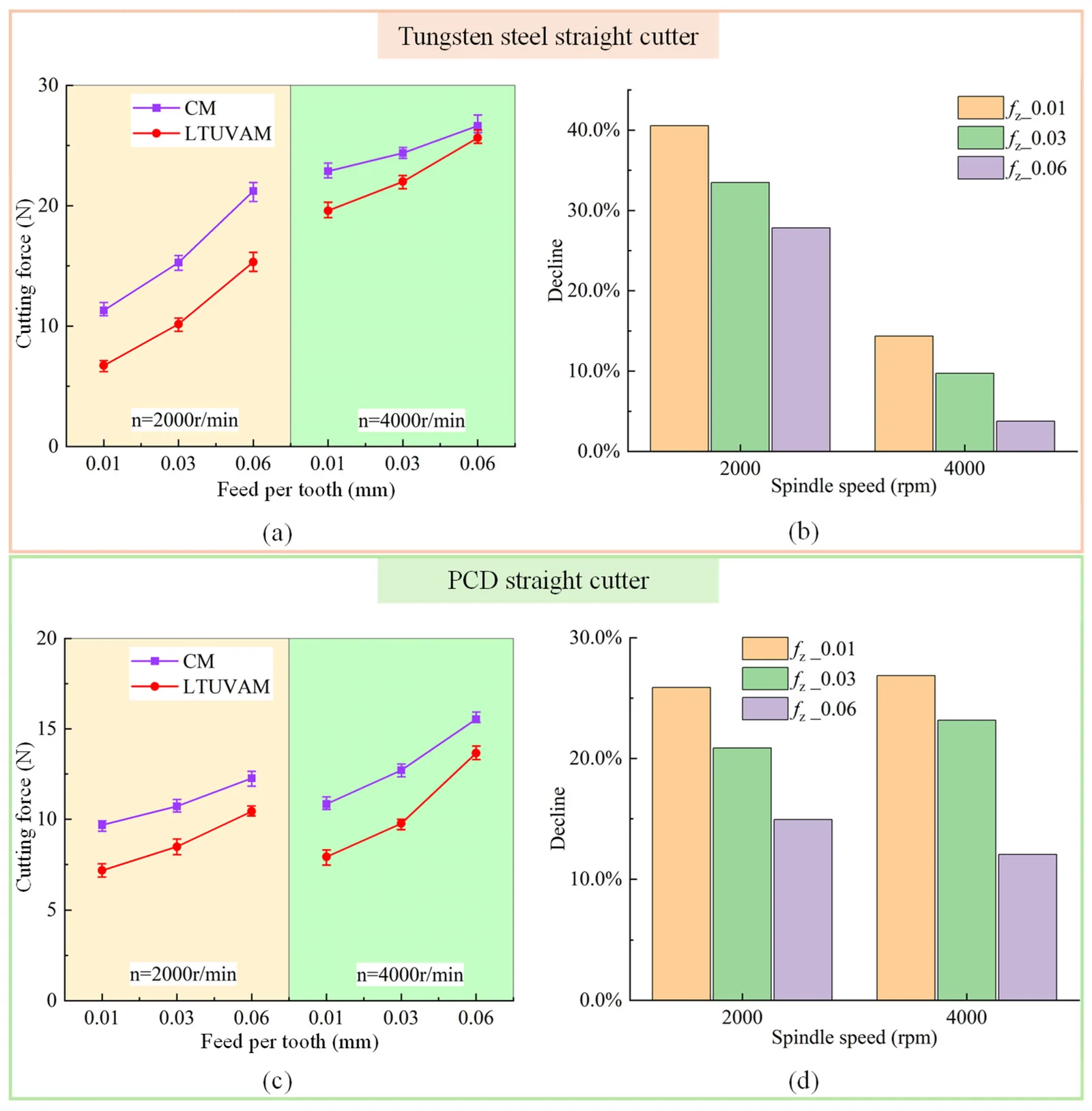
(**a**) The average values of experimental forces and (**b**) the reduction of the cutting forces in LTUVAM processes compared to CM processes utilizing a tungsten steel straight cutter; (**c**) the average values of cutting forces and (**d**) the reduction ratio of the cutting forces under LTUVAM conditions compared to CM conditions utilizing a PCD straight cutter.

Figure 10a presents the average values of experimental cutting forces under different machining conditions utilizing a tungsten steel straight cutter; Figure 10b provides the reduction ratio of the tungsten steel straight cutter cutting forces in LTUVAM processes compared to CM processes; Figure 10c demonstrates the average milling forces for different machining parameters utilizing a PCD straight cutter; and Figure 10d illustrates the reduction ratio of the PCD cutter cutting forces under LTUVAM conditions compared to CM conditions.

Compared to CM operations, LTUVAM could reduce cutting forces under all the conditions outlined in Figure 10. However, the reduction rates of cutting forces reveal a clear dependence on machining parameters. As the feed per tooth increases, the reduction rate of cutting forces achieved through LTUVAM tends to decrease, indicating that the suppressive effect of LTUVAM on cutting forces is more significant under small feed machining conditions.

**Figure 11:**
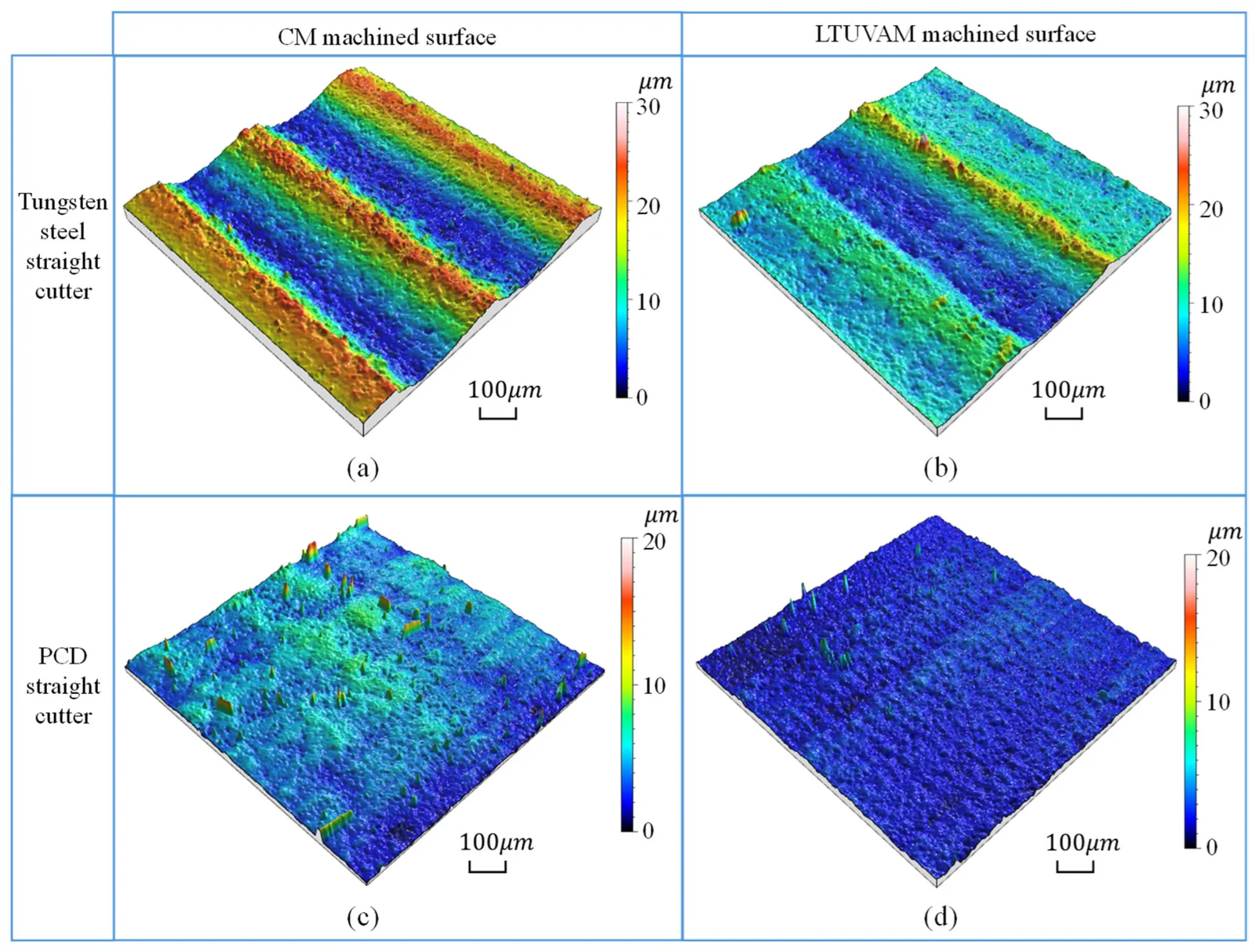
The machined surface measurements for (**a**) CM and (**b**) LTUVAM utilizing a tungsten steel straight cutter; the PCD straight cutter machined surfaces for (**c**) CM and (**d**) LTUVAM conditions.

Furthermore, the examples of machined surfaces could also directly demonstrate the effectiveness of LTUVAM in surface quality improvement. Figure 11a provides the tungsten steel straight cutter machined surface topography after CM; it can be observed that the machined surface is in poor condition, with deep tool marks remaining. Figure 11b demonstrates that substantially better surface quality can be achieved by the LTUVAM; however, there are still some tool marks left. To further improve the machined surface quality, a PCD straight cutter, which could offer higher hardness, a lower coefficient of friction, and greater machining accuracy, was utilized to mill the composite workpiece. Figure 11c demonstrates the PCD cutter machined surface after the CM process, indicating that this tool produces better surface topography than a tungsten carbide tool. To validate the effectiveness of LTUVAM on the PCD cutter, a machining trial was conducted; a more satisfactory machined surface (see Figure 11d) was achieved compared to CM. These four milling trials were conducted under the same condition: 2000 rpm spindle speed, 0.06 mm feed per tooth, 4 mm axial depth of cut, and 1 mm radial depth of cut. It can be concluded that compared to CM, LTUVAM produces a better surface finish with both tungsten steel and PCD straight cutters. Also, the machining surfaces produced by the PCD tool are significantly better than those produced by the tungsten steel tool.

## 5. Conclusions

A finite element analysis method for LTUVAM of UD-CF/PPS processes is provided in this paper. The kinematic analysis of the LTUVAM and the mechanism of surface formation during UD-CF/PPS milling process are proposed, enabling the capability of simultaneously simulating the longitudinal and torsional motions of the cutter in UD-CF/PPS ultrasonic vibration-assisted milling. During the verification, simulated cutting forces showed satisfactory agreement with experimental forces, and the LTUVAM produced decreased cutting forces and superior machined surface quality compared to CM. Three different cutters were employed to validate the effectiveness of LTUVAM under different helix angles and materials of the tool; at the microscopic level, fiber debonding was observed from the CM process, and it could have been eliminated in the LTUVAM process. At the macroscopic level, the height of machined surfaces could be significantly reduced by LTUVAM. Also, the experimental investigation suggested that the suppressive effect of LTUVAM on cutting forces is more significant under small feed machining conditions.

The findings proposed in this paper indicate that the developed approach could be utilized to simulate LTUVAM of CF/PPS materials, and the longitudinal torsional ultrasonic vibration technology could offer a greater milling result than CM operation. Furthermore, this research allows not only the improvement of the CF/PPS milling process through LTUVAM but also the possibility of the following objectives: cutting parameters optimization, cutting tool selection, and modeling of the UD-CF/PPS drilling process.

This study also has some limitations; further research could focus on these areas:

(1) This study was limited to unidirectional fiber composites; further research is needed on multidirectional fiber composites.
(2) The findings in this study indicate that the benefit of the LTUVAM (such as force reduction and surface quality improvement) is more effective at lower spindle speed. This is because at lower spindle speed, the ultrasonic vibration speed can be higher than the cutting speed, resulting in the intermittent separation phenomenon between the tool and workpiece; this phenomenon generally leads to the reduction in cutting force and improves surface quality. However, it should be noted that this does not indicate that high spindle speed cannot be selected during the LTUVAM process. Since the ultrasonic vibration velocity is positively correlated with ultrasound frequency and vibration amplitude, if a higher spindle speed is needed, the effects of LTUVAM can still be achieved by increasing the ultrasonic frequency or the amplitude of vibration.

## Figures and Tables

**Table 1:** The measured longitudinal torsional ultrasonic parameters.

Types of Cutting Tools	Tool Diameter	Ultrasound Power	Ultrasound Frequency	Vertical Amplitude	Torsional Amplitude
Helical milling cutter	6 mm	100%	19.9 kHz	5.31 μm	5.1 μm
Tungsten steel straight cutter	6 mm	100%	19.9 kHz	2.89 μm	10.53 μm
PCD straight cutter	6 mm	100%	19.9 kHz	5.19 μm	5.28 μm

## Data Availability

The data presented in this study are available within the article.
